# Supplementary material for: Progression-Dependent Altered Metabolism in Osteosarcoma Resulting in Different Nutrient Source Dependencies
Source: Cancers (Basel). 2020 May 27;12(6):1371. doi: 10.3390/cancers12061371 (PMC7352851; doi:10.3390/cancers12061371)
Supplement: Supplementary file 1 [file cancers-12-01371-s001.pdf]

Supplementary Materials

Progression-Dependent Altered Metabolism in Osteosarcoma Resulting in Different Nutrient Source Dependencies

Raphaela Fritsche-Guenther, Yoann Gloaguen, Marieluise Kirchner, Philipp Mertins, Per-Ulf Tunn and Jennifer A. Kirwan

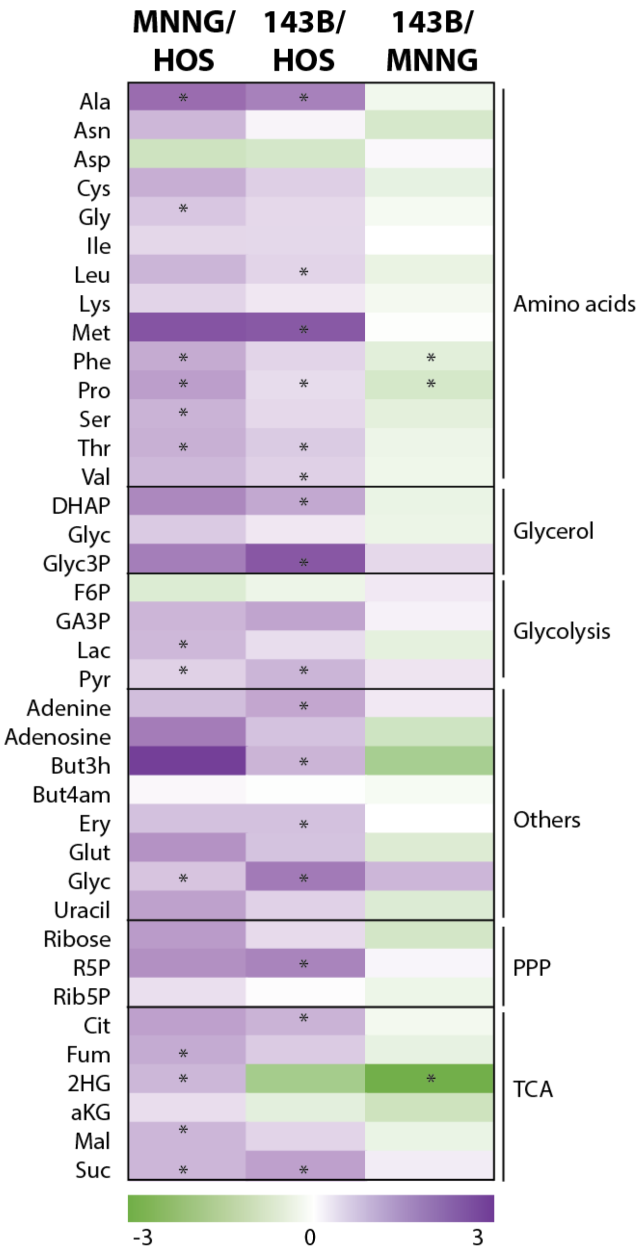

Figure S1. Heat map from the annotated metabolites comparing MNNG/HOS to HOS, 143B to HOS and 143B to MNNG/HOS cells. For each $n = 3$ biological and $n = 2$ technical replicates measured. Log2 values are shown from the ratios calculated from the mean of the normalized peak areas from the replicates. PPP: Pentose phosphate pathway. TCA: Tricarboxylic acid. Samples were analyzed using an unpaired Student's t-test with $p < 0.05$ deemed as statistically significant (indicated with the star). Abbreviations see Table S5.

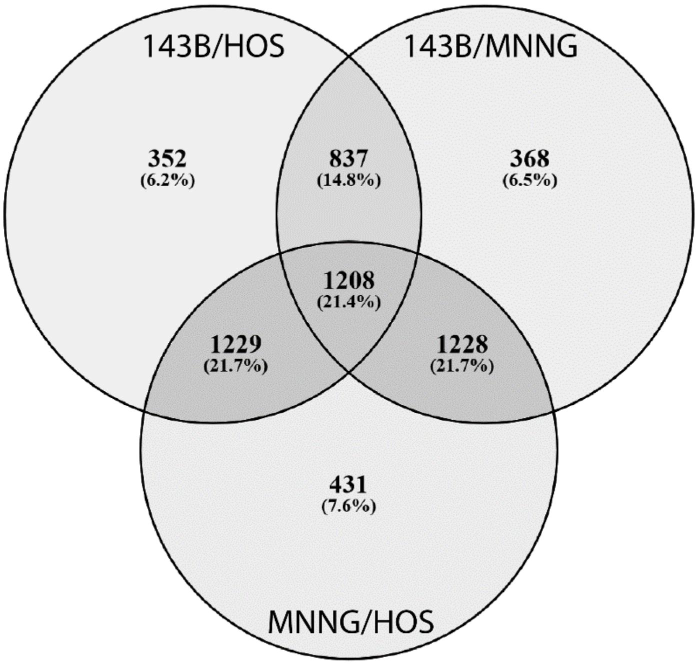

Figure S2. Venn diagram showing the number of proteins which are significantly differentially expressed between HOS, MNNG/HOS (MNNG) or 143B cells (Created using Venny (https://bioinfogp.cnb.csic.es/tools/venny/index.html)). $n = 5$ replicates were measured.

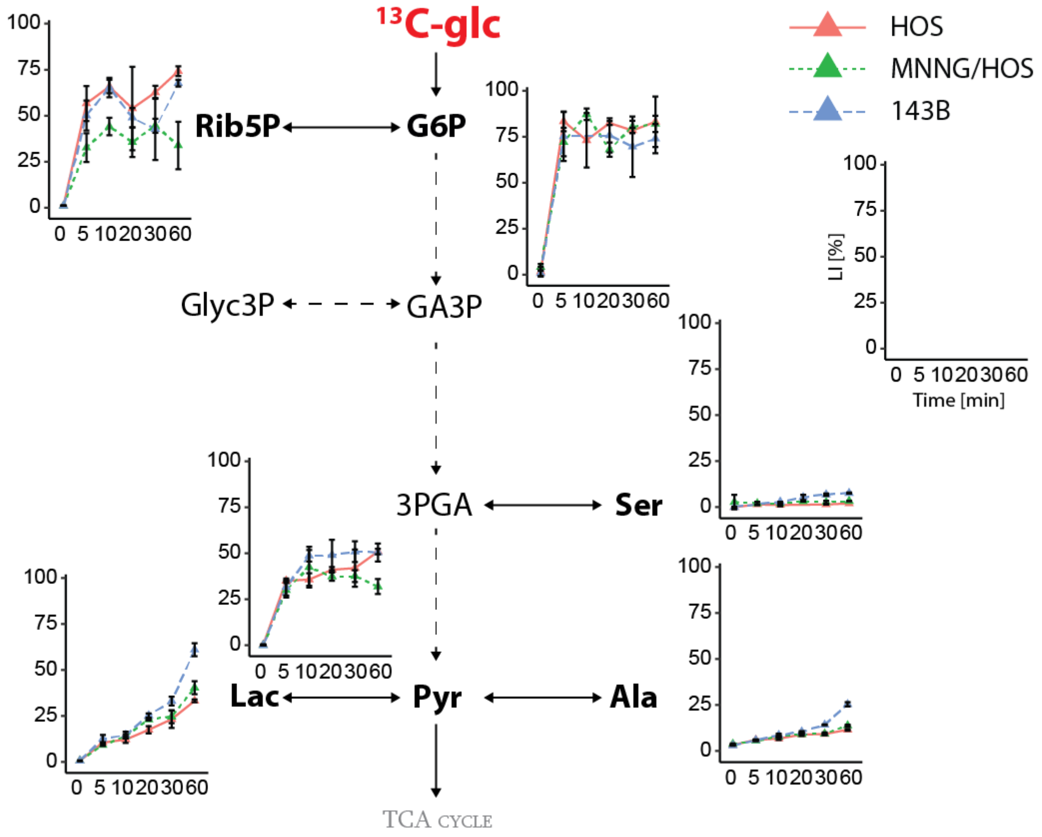

Figure S3. Levels of label incorporation (LI) of glycolytic intermediates in osteosarcoma cells ($n = 3$ replicates) in the presence of ^{13}C-glucose. Abbreviations see Figure 1.

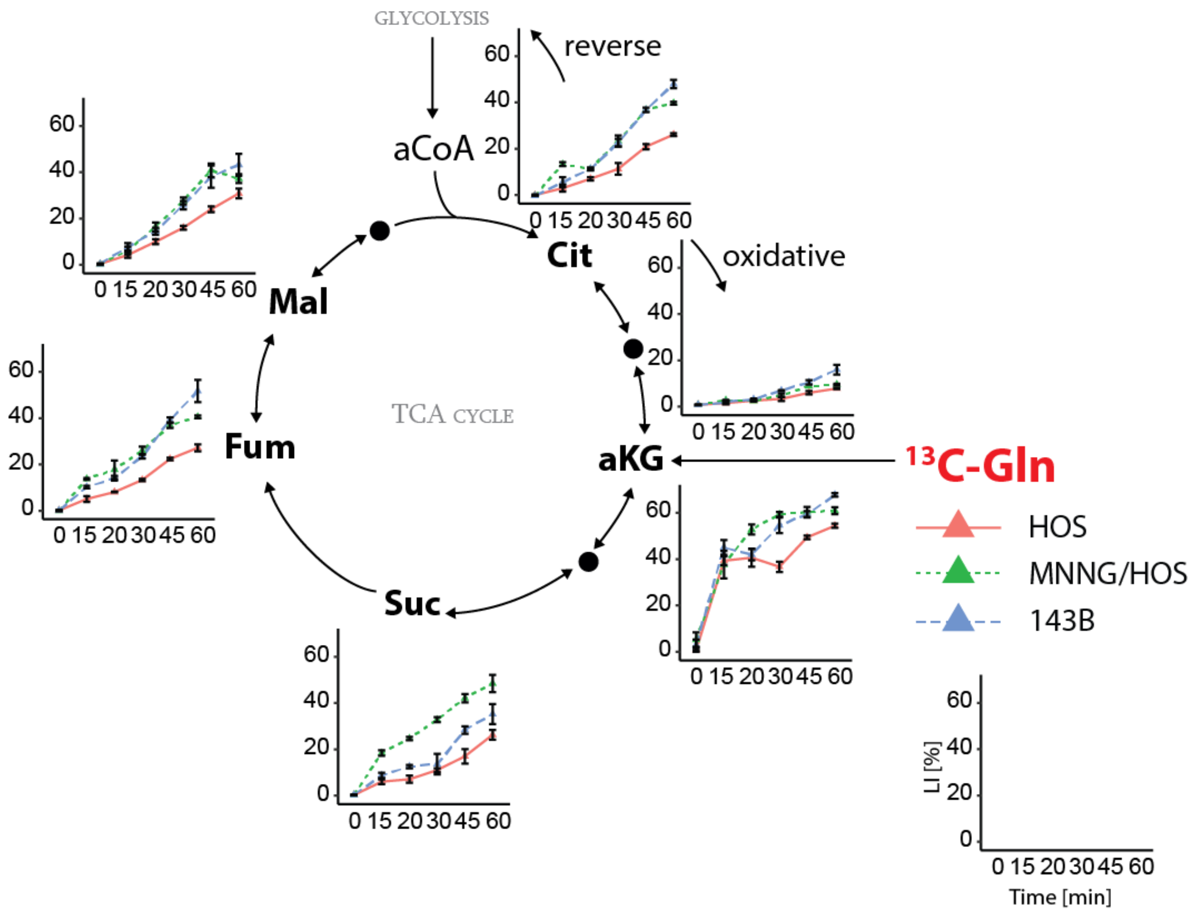

Figure S4. Levels of label incorporation (LI) of glycolytic intermediates in osteosarcoma cells (n = 3 replicates) in the presence of ^{13}C-glutamine. Abbreviations see Figure 1.

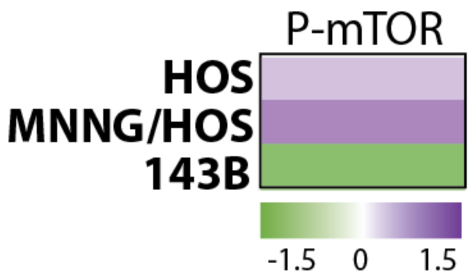

Figure S5. Heat map of phosphorylated mTOR (S2448) comparing 24 h and 0 h of treatment with 3-Bromopyruvic acid (BrPy, 0.25 mM) in osteosarcoma cell lines. Shown are log2 values from the ratios calculated from the mean fluorescence intensity from the biological replicates (n = 3).

Table S1. Mean and standard deviation (SD) of the normalized peak area from the annotated central carbon metabolites in *n* = 3 biological and *n* = 2 technical replicates of HOS, MNNG/HOS and 143B cells using a gas chromatography mass spectrometry (GC-MS) approach. AA: Amino acid. PPP: Pentose phosphate pathway. TCA: Tricarboxylic acid.

Metabolite	Group	Mean			SD		
		HOS	MNNG/ HOS	143B	HOS	MNNG/ HOS	143B
Alanine	AA	0.364	1.349	1.298	0.118	0.542	0.180
Asparagine	AA	0.004	0.007	0.005	0.002	0.002	0.002
Aspartic acid	AA	0.124	0.055	0.067	0.149	0.027	0.038
Glycine	AA	0.362	0.549	0.543	0.083	0.167	0.021
Isoleucine	AA	0.007	0.009	0.010	0.002	0.001	0.004
Leucine	AA	0.130	0.236	0.203	0.025	0.100	0.014
Lysine	AA	0.030	0.038	0.038	0.013	0.015	0.015
Methionine	AA	0.005	0.029	0.028	0.001	0.012	0.002
Phenylalanine	AA	0.035	0.070	0.054	0.013	0.019	0.003
Proline	AA	0.273	0.625	0.395	0.057	0.185	0.027
Serine	AA	0.148	0.272	0.219	0.039	0.095	0.018
Threonine	AA	0.085	0.163	0.146	0.021	0.045	0.005
Valine	AA	0.070	0.122	0.114	0.019	0.045	0.005
Dihydroxyacetone phosphate	Glycerol	0.000	0.001	0.001	0.000	0.000	0.000
Glycerol	Glycerol	0.440	0.615	0.571	0.107	0.076	0.067
Glycerol-3-phosphate	Glycerol	0.002	0.008	0.014	0.001	0.003	0.003
Fructose-6-phosphate	Glycolysis	0.020	0.013	0.015	0.005	0.007	0.015
Glyceric acid-3-phosphate	Glycolysis	0.003	0.004	0.007	0.001	0.004	0.002
Lactic acid	Glycolysis	2.190	3.724	3.121	0.588	1.333	0.210
Pyruvic acid	Glycolysis	0.011	0.015	0.023	0.003	0.004	0.003
Adenine	Others	0.001	0.002	0.003	0.000	0.001	0.000
Adenosine	Others	0.001	0.002	0.001	0.000	0.001	0.000
Butanoic acid, 3-hydroxy	Others	0.002	0.014	0.004	0.001	0.019	0.001
Butanoic acid, 4-amino	Others	0.004	0.004	0.004	0.001	0.001	0.001
Cytosine	Others	0.016	0.033	0.027	0.007	0.008	0.000
Erythritol	Others	0.022	0.033	0.041	0.007	0.011	0.003
Glutaric acid	Others	0.001	0.003	0.002	0.001	0.002	0.001
Glyceric acid	Others	0.003	0.004	0.011	0.001	0.001	0.003
Ribose	Others	0.001	0.003	0.002	0.001	0.002	0.000
Uracil	Others	0.013	0.023	0.021	0.011	0.003	0.010
Ribose-5-phosphate	PPP	0.001	0.003	0.004	0.000	0.002	0.001
Ribulose-5-phosphate	PPP	0.003	0.004	0.003	0.002	0.002	0.002
Citric acid	TCA	0.032	0.067	0.069	0.008	0.006	0.005
Fumaric acid	TCA	0.016	0.033	0.028	0.007	0.007	0.005
Glutaric acid, 2-hydroxy	TCA	0.004	0.008	0.001	0.002	0.002	0.000
Glutaric acid, 2-oxo	TCA	0.004	0.004	0.002	0.003	0.001	0.000
Malic acid	TCA	0.046	0.082	0.071	0.013	0.025	0.003
Succinic acid	TCA	0.010	0.017	0.026	0.003	0.006	0.001

Table S2. Mean and standard deviation (SD) of the normalized peak area/intensity from the compounds in *n* = 5 HOS, MNNG/HOS and 143B cells using the AbsoluteIDQ p400 HR kit (Biocrates). Shown are the compounds measured with the flow injection (FIA) and liquid chromatography (LC) method. CE: cholesterylesters. DG: diglycerides. H1: hexoses. LPC: lysophosphatidylcholines. NA: not available. PC: phosphatidylcholines. SM: sphingomyelins. TG: triglycerides.

**Compound**	**Mean**			**SD**		
	**HOS**	**MNNG/HOS**	**143B**	**HOS**	**MNNG/HOS**	**143B**
DG-O (34:1)	0.066	0.122	0.110	0.016	0.026	0.022
DG (32:1)	0.041	0.056	0.036	0.009	0.011	0.005
DG (34:1)	0.081	0.154	0.118	0.032	0.039	0.020
DG (36:2)	0.034	0.060	0.050	0.006	0.009	0.012
LPC (18:1)	0.026	0.083	0.047	0.007	0.024	0.011
LPC (20:4)	0.021	0.068	0.032	0.005	0.015	0.009
PC-O (30:0)	0.035	0.076	0.087	0.014	0.015	0.019
PC-O (32:0)	0.168	0.297	0.262	0.072	0.069	0.052
PC-O (32:1)	0.253	0.632	0.433	0.098	0.079	0.095
PC-O (32:2)	0.047	0.054	0.057	0.014	0.005	0.013
PC-O (32:3)	0.022	0.044	0.040	0.007	0.009	0.007
PC-O (34:0)	0.031	0.098	0.089	0.022	0.043	0.020
PC-O (34:1)	0.245	0.751	0.486	0.110	0.316	0.105
PC-O (34:2)	0.084	0.178	0.152	0.052	0.023	0.036
PC-O (34:3)	0.037	0.080	0.055	0.016	0.032	0.012
PC-O (34:4)	0.041	0.135	0.081	0.018	0.019	0.020
PC-O (36:1)	0.026	0.129	0.181	0.016	0.038	0.044
PC-O (36:2)	0.051	0.175	0.162	0.021	0.028	0.039
PC-O (36:3)	0.042	0.142	0.081	0.026	0.019	0.018
PC-O (36:4)	0.097	0.383	0.200	0.035	0.063	0.048
PC-O (36:5)	0.026	0.118	0.066	0.014	0.014	0.016
PC-O (38:1)	0.005	0.008	0.016	0.001	0.002	0.004
PC-O (38:2)	0.009	0.016	0.021	0.004	0.003	0.005
PC-O (38:3)	0.021	0.040	0.026	0.010	0.011	0.006
PC-O (38:5)	0.042	0.156	0.096	0.022	0.016	0.023
PC-O (38:6)	0.032	0.113	0.048	0.015	0.010	0.011
PC-O (40:3)	0.006	NA	0.005	0.002	NA	0.001
PC-O (40:4)	0.009	0.009	0.011	0.004	0.002	0.003
PC-O (40:5)	0.011	0.026	0.022	0.005	0.006	0.005
PC-O (40:6)	0.018	0.046	0.029	0.011	0.005	0.007
PC-O (40:7)	0.025	0.048	0.025	0.012	0.004	0.007
PC-O (40:8)	0.018	0.041	0.015	0.007	0.004	0.004
PC (29:0)	NA	0.014	0.023	NA	0.002	0.005
PC (30:0)	0.134	0.203	0.325	0.043	0.030	0.065
PC (30:1)	0.068	0.147	NA	0.029	0.013	NA
PC (31:0)	0.022	0.016	0.040	0.004	0.005	0.009
PC (31:1)	0.026	NA	NA	0.012	NA	NA
PC (32:0)	0.135	0.149	0.226	0.045	0.029	0.053
PC (32:1)	0.580	0.967	0.787	0.468	0.414	0.042
PC (32:2)	0.083	0.058	0.028	0.059	0.030	0.005
PC (32:3)	0.081	0.113	0.143	0.025	0.017	0.022
PC (32:4)	0.029	0.069	0.042	0.012	0.007	0.006
PC (33:0)	0.008	0.010	0.016	0.002	0.002	0.004
PC (33:1)	0.045	NA	0.105	0.024	NA	0.031
PC (33:2)	0.020	0.023	0.024	0.017	0.010	0.004
PC (33:4)	0.008	0.012	0.009	0.003	0.001	0.002
PC (34:0)	0.028	0.013	0.014	0.015	0.003	0.001
PC (34:1)	1.115	1.815	1.930	0.524	0.289	0.381
PC (34:2)	0.444	0.569	0.414	0.336	0.129	0.041
PC (34:3)	0.078	0.105	0.103	0.029	0.030	0.021
PC (34:4)	0.134	0.354	0.275	0.078	0.036	0.058

PC (34:5)	0.023	0.030	0.014	0.011	0.003	0.003
PC (35:1)	0.017	0.046	0.064	0.006	0.009	0.012
PC (35:2)	0.027	0.062	0.049	0.011	0.009	0.008
PC (35:3)	0.007	0.008	0.010	0.003	0.001	0.003
PC (35:4)	0.022	0.054	0.049	0.016	0.005	0.010
PC (36:1)	0.103	0.393	0.577	0.049	0.077	0.133
PC (36:2)	0.498	1.264	0.996	0.189	0.139	0.222
PC (36:3)	0.121	0.256	0.181	0.051	0.049	0.036
PC (36:4)	0.376	1.098	0.820	0.172	0.251	0.180
PC (36:5)	0.109	0.291	0.152	0.067	0.027	0.033
PC (36:6)	0.014	0.017	0.008	0.007	0.001	0.001
PC (37:2)	0.012	0.033	0.034	0.004	0.004	0.008
PC (37:4)	0.016	0.051	0.045	0.009	0.011	0.009
PC (37:5)	0.018	0.043	0.025	0.010	0.003	0.005
PC (38:2)	0.046	0.146	0.177	0.017	0.018	0.041
PC (38:3)	0.054	0.073	0.074	0.022	0.007	0.014
PC (38:4)	0.101	0.291	0.295	0.053	0.069	0.062
PC (38:5)	0.223	0.762	0.493	0.120	0.074	0.102
PC (38:6)	0.053	0.128	0.078	0.033	0.015	0.017
PC (38:7)	0.019	0.031	0.018	0.009	0.003	0.004
PC (39:3)	0.010	0.013	0.010	0.004	0.002	0.001
PC (39:4)	0.007	0.010	0.009	0.003	0.001	0.002
PC (40:2)	0.007	0.017	0.028	0.002	0.003	0.006
PC (40:3)	0.011	0.013	0.017	0.003	0.002	0.003
PC (40:4)	0.021	0.035	0.035	0.012	0.003	0.006
PC (40:5)	0.030	0.092	0.095	0.018	0.009	0.020
PC (40:6)	0.038	0.069	0.053	0.023	0.007	0.011
PC (40:7)	0.041	0.070	0.044	0.020	0.006	0.010
PC (40:8)	0.015	0.033	0.023	0.010	0.003	0.005
PC (40:9)	0.012	0.028	0.017	0.004	0.002	0.004
PC (42:10)	0.011	0.030	0.018	0.007	0.001	0.006
PC (42:5)	0.008	0.012	0.015	0.003	0.003	0.003
PC (42:6)	0.008	0.008	0.011	0.004	0.001	0.002
PC (42:7)	0.012	0.009	0.009	0.006	0.001	0.002
PC (44:10)	0.006	NA	NA	0.002	NA	NA
SM (32:1)	0.016	0.021	0.024	0.004	0.003	0.005
SM (33:1)	0.013	0.016	0.027	0.003	0.002	0.007
SM (34:1)	0.252	0.312	0.570	0.084	0.063	0.150
SM (34:2)	0.026	0.032	0.029	0.006	0.003	0.007
SM (40:1)	0.012	NA	0.017	0.003	NA	0.004
SM (40:2)	0.006	0.007	0.009	0.002	0.001	0.002
SM (42:1)	0.024	0.043	0.069	0.006	0.020	0.016
SM (42:2)	0.079	0.071	0.129	0.020	0.019	0.030
SM (42:3)	0.015	0.016	0.015	0.004	0.003	0.003
DG-O (36:4)	NA	0.066	0.052	NA	0.007	0.008
H1	NA	0.089	NA	NA	0.008	NA
LPC (16:0)	NA	0.056	0.059	NA	0.008	0.009
LPC (18:0)	NA	0.006	0.008	NA	0.001	0.002
LPC (20:3)	NA	0.012	0.009	NA	0.002	0.002
PC-O (30:1)	NA	0.027	0.032	NA	0.006	0.008
PC-O (33:2)	NA	0.004	0.005	NA	0.000	0.001
PC-O (35:3)	NA	0.006	0.004	NA	0.001	0.001
PC-O (35:4)	NA	0.016	NA	NA	0.003	NA
PC-O (36:6)	NA	0.009	0.006	NA	0.001	0.002
PC-O (38:4)	NA	0.134	0.098	NA	0.035	0.022
PC (32:5)	NA	0.018	0.013	NA	0.005	0.002
PC (33:3)	NA	0.010	0.011	NA	0.003	0.002
PC (35:0)	NA	0.005	0.006	NA	0.001	0.001
PC (37:1)	NA	0.006	0.009	NA	0.000	0.002

PC (37:3)	NA	0.010	0.010	NA	0.002	0.002
PC (37:6)	NA	0.006	NA	NA	0.001	NA
PC (38:1)	NA	0.037	0.053	NA	0.005	0.010
PC (39:2)	NA	0.007	0.007	NA	0.001	0.001
PC (39:5)	NA	0.019	0.017	NA	0.002	0.003
PC (39:6)	NA	0.007	0.006	NA	0.000	0.001
PC (40:1)	NA	0.007	0.011	NA	0.002	0.002
PC (44:5)	NA	0.005	0.008	NA	0.001	0.002
SM (36:1)	NA	0.004	0.014	NA	0.002	0.002
CE (22:5)	NA	NA	0.251	NA	NA	0.097
CE (22:6)	NA	NA	0.436	NA	NA	0.212
PC-O (40:2)	NA	NA	0.007	NA	NA	0.002
PC-O (42:2)	NA	NA	0.005	NA	NA	0.001
PC-O (42:5)	NA	NA	0.006	NA	NA	0.001
PC-O (44:4)	NA	NA	0.006	NA	NA	0.001
PC (42:1)	NA	NA	0.009	NA	NA	0.003
PC (42:4)	NA	NA	0.009	NA	NA	0.002
PC (44:1)	NA	NA	0.010	NA	NA	0.002
PC (46:2)	NA	NA	0.005	NA	NA	0.001
SM (41:1)	NA	NA	0.006	NA	NA	0.001
SM (41:2)	NA	NA	0.005	NA	NA	0.001
TG (48:1)	NA	NA	0.017	NA	NA	0.008
TG (50:2)	NA	NA	0.021	NA	NA	0.013
TG (52:2)	NA	NA	0.032	NA	NA	0.023
ADMA	NA	NA	0.015	NA	NA	0.004
Alanine	1.022	1.977	3.840	0.098	0.288	0.683
Arginine	0.191	0.692	0.163	0.074	0.120	0.036
Asparagine	0.267	0.756	0.981	0.031	0.069	0.175
Aspartic acid	0.067	0.201	0.259	0.014	0.020	0.079
Citrullin	NA	NA	0.029	NA	NA	0.008
Creatinine	0.054	0.059	0.063	0.011	0.006	0.004
Glutamine	0.072	0.014	0.030	0.033	0.001	0.004
Glutamic acid	1.189	1.093	2.762	0.206	0.127	0.657
Glycine	0.033	0.096	0.079	0.005	0.011	0.016
Histamine	0.031	0.069	0.143	0.003	0.009	0.027
Isoleucine	0.120	0.201	0.427	0.022	0.024	0.071
Lysine	0.034	0.096	0.028	0.012	0.011	0.007
Methionine	0.008	0.012	0.011	0.001	0.002	0.003
Methionine-SO	NA	0.009	NA	NA	0.001	NA
Ornithine	0.159	0.084	0.546	0.020	0.008	0.086
Phenylalanine	0.015	0.027	0.024	0.002	0.003	0.005
Proline	0.255	0.307	2.406	0.038	0.040	0.476
Putrescine	0.664	1.965	0.078	0.141	0.460	0.033
Serine	0.037	0.094	0.097	0.010	0.010	0.027
Spermidine	8.237	13.694	16.627	1.160	1.802	3.023
Spermine	1.294	0.673	0.772	0.585	0.086	0.455
t4-OH-Pro	0.915	1.701	4.123	0.071	0.312	0.602
Taurine	0.120	0.206	0.265	0.015	0.016	0.060
Threonine	0.052	0.134	0.134	0.002	0.011	0.029
Tryptophan	0.004	0.010	0.015	0.001	0.003	0.003
Tyrosine	0.041	0.089	0.141	0.005	0.010	0.025
Valine	0.032	0.055	0.040	0.008	0.003	0.010
Leucine	0.306	0.530	1.004	0.033	0.089	0.133

Table S3. Proliferation of OS cells after treatment with 0.25 mM 3-Bromopyruvic acid (BrPy) or PBS solvent control in *n* = 3 HOS, MNNG/HOS and 143B cells. Time point 0 hours (h) proliferation value was set to 0% to calculate the percentage of proliferation over time.

Time	Treatment	HOS	MNNG/HOS	143B
0 h	Control	0	0	0
24 h	Control	100	184	222
48 h	Control	422	501	393
72 h	Control	386	672	335
0 h	BrPy	0	0	0
24 h	BrPy	−27	10	−44
48 h	BrPy	5	35	−40
72 h	BrPy	−57	245	−59

Table S4. Mean and standard deviation (SD) of the normalized peak area from the annotated central carbon metabolites in osteosarcoma *n* = 7 primary tumors (PT) and *n* = 5 lung metastases (LM) using a gas chromatography mass spectrometry (GC-MS) approach. M: Male. F: Female. AA: Amino acid. PPP: Pentose phosphate pathway. TCA: Tricarboxylic acid.

Metabolites	Group	Mean			SD		
		PT	LM		PT	LM	
		M + F	Male	Female	M + F	Male	Female
Alanine	AA	10.03	14.07	2.22	6.56	11.09	0.70
Asparagine	AA	0.01	0.03	0.01	0.00	0.03	0.00
Aspartic acid	AA	1.14	4.38	0.09	0.75	1.31	0.06
Cysteine	AA	0.39	0.48	0.11	0.17	0.35	0.01
Glycine	AA	9.39	9.81	2.26	5.32	3.37	0.97
Isoleucine	AA	0.01	0.00	0.00	0.00	0.00	0.00
Leucine	AA	0.61	0.72	0.18	0.18	0.31	0.06
Lysine	AA	0.40	0.42	0.15	0.27	0.14	0.10
Methionine	AA	0.03	0.03	0.02	0.01	0.00	0.01
Phenylalanine	AA	0.62	0.70	0.08	0.24	0.58	0.03
Proline	AA	2.68	2.66	1.05	1.35	1.20	0.25
Serine	AA	0.37	0.45	0.09	0.21	0.09	0.06
Threonine	AA	0.33	0.50	0.11	0.18	0.05	0.04
Tryptophan	AA	0.13	0.11	0.03	0.04	0.06	0.02
Tyrosine	AA	0.06	0.03	0.02	0.04	0.02	0.00
Valine	AA	1.81	1.76	0.47	0.35	0.78	0.17
Glycerol	Glycerol	6.48	9.35	1.93	3.25	8.07	0.35
Glycerol-3-phosphate	Glycerol	0.23	0.46	0.03	0.25	0.33	0.01
Phosphoenolpyruvic acid	Glycolysis	0.15	0.32	0.03	0.11	0.03	0.01
Pyruvic acid	Glycolysis	0.12	0.14	0.07	0.07	0.04	0.08
Lactic acid	Glycolysis	109.88	137.52	54.21	27.92	7.87	15.84
Adenine	Others	0.06	0.05	0.01	0.03	0.03	0.00
Butanoic acid, 3-hydroxy-	Others	4.32	2.24	0.48	5.99	1.57	0.35
Butanoic acid, 4-amino-	Others	0.04	0.02	0.01	0.04	0.02	0.01
Cytosine	Others	0.08	0.08	0.02	0.02	0.03	0.01
Erythritol	Others	0.12	0.09	0.04	0.12	0.03	0.00
Glutaric acid	Others	0.02	0.02	0.01	0.01	0.01	0.01
Glyceric acid	Others	0.13	0.03	0.03	0.13	0.00	0.01
Ribose	Others	0.46	0.06	0.18	0.49	0.03	0.10
Uracil	Others	0.79	0.39	0.26	0.39	0.11	0.10
Ribose-5-phosphate	PPP	0.02	0.03	0.02	0.03	0.01	0.02
Citric acid	TCA	0.50	0.37	0.08	0.21	0.03	0.05
Fumaric acid	TCA	0.12	0.17	0.07	0.04	0.03	0.01
Malic acid	TCA	0.39	0.69	0.04	0.29	0.27	0.02
Succinic acid	TCA	1.95	2.27	0.20	1.51	0.47	0.12

Table S5. List of metabolite derivatives and their biological group used for reference search. AA: Amino acids. PPP: Pentose phosphate pathway. TCA: Tricarboxylic acid cycle. TMS: Trimethylsilyl derivatives. MeOX: Methoxyamine hydrochloride.

Group	Metabolite	Abbreviation	Detected as
AA	Alanine	Ala	3TMS
			2TMS
AA	Asparagine	Asn	2TMS
AA	Aspartic acid	Asp	2TMS
			3TMS
AA	Cysteine	Cys	3TMS
AA	Glycine	Gly	2TMS
			3TMS
AA	Isoleucine	Ile	1TMS
			2TMS
AA	Leucine	Leu	1TMS
			2TMS
AA	Lysine	Lys	3TMS
AA	Methionine	Met	1TMS
			2TMS
AA	Phenylalanine	Phe	1TMS
			2TMS
AA	Proline	Pro	1TMS
			2TMS
AA	Serine	Ser	2TMS
			3TMS
			4TMS
AA	Threonine	Thr	2TMS
			3TMS
AA	Tryptophan	Trp	2TMS
AA	Tyrosine	Tyr	3TMS
AA	Valine	Val	1TMS
			2TMS
Glycerol	Dihydroxyacetone phosphate	DHAP	1MeOX 3TMS
Glycerol	Glycerol	Glyc	3TMS
Glycerol	Glycerol-3-phosphate	Glyc3P	4TMS
Glycolysis	Fructose-6-phosphate	F6P	1MeOX 6TMS
Glycolysis	Glucose-6-phosphate	G6P	1MeOX 6TMS
Glycolysis	Glyceric acid-3-phosphate	GA3P	4TMS
Glycolysis	Lactic acid	Lac	2TMS
Glycolysis	Phosphoenolpyruvic acid	PEP	3TMS
Glycolysis	Pyruvic acid	Pyr	1MeOX 1TMS
Nucleobase	Adenine	Adenine	2TMS
Nucleobase	Uracil	Uracil	2TMS
Nucleosid	Adenosine	Adenosine	3TMS
			4TMS
Nucleosid	Cytosine	Cytosine	2TMS
Others	Butanoic acid, 3-hydroxy-	But3h	2TMS
Others	Butanoic acid, 4-amino-	But4am	3TMS
Others	Erythritol	Ery	4TMS
Others	Glutaric acid	Glut	2TMS
Others	Glyceric acid	Glyc	3TMS
PPP	Ribose-5-phosphate	R5P	1MeOX 5TMS
PPP	Ribose	Ribose	1MeOX 4TMS
TCA	Citric acid	Cit	4TMS
TCA	Fumaric acid	Fum	2TMS
TCA	Glutaric acid, 2-hydroxy-	2HG	3TMS
TCA	Glutaric acid, 2-oxo-	aKG	1MeOX 2TMS
TCA	Malic acid	Mal	3TMS
TCA	Succinic acid	Suc	2TMS

Table S6. Technical variation during gas chromatography mass spectrometry (GC-MS) runs. RSD: Relative standard deviation. QC: quality control. Glc: glucose. Gln: glutamine.

Parameter	Patients QC	Cell Lines QC	^{13}C-glc QC	^{13}C-gln QC
Replicates	5	6	5	5
RSD of sum of normalized peak area	11%	5%	33%	26%
RSD of Alkane 32	11%	13%	14%	10%
RSD of internal standard	17%	6%	12%	18%
Median RSD individual metabolites	28%	20%	45%	57%

© 2020 by the authors. Licensee MDPI, Basel, Switzerland. This article is an open access article distributed under the terms and conditions of the Creative Commons Attribution (CC BY) license (http://creativecommons.org/licenses/by/4.0/).
